# Staphylococcus aureus in Substrates for Black Soldier Fly Larvae (Hermetia illucens) and Its Dynamics during Rearing

**DOI:** 10.1128/spectrum.02183-21

**Published:** 2021-12-22

**Authors:** E. Gorrens, N. Van Looveren, L. Van Moll, D. Vandeweyer, D. Lachi, J. De Smet, L. Van Campenhout

**Affiliations:** a Department of Microbial and Molecular Systems (M2S), Research Group for Insect Production and Processing, KU Leuven, Geel Campus, Geel, Belgium; b Leuven Food Science and Nutrition Research Centre (LFoRCe), KU Leuven, Leuven, Belgium; c Faculty of Pharmaceutical, Biomedical and Veterinary Sciences, Laboratory for Microbiology, Parasitology and Hygiene (LMPH), University of Antwerp, Wilrijk, Belgium; University of Minnesota

**Keywords:** *Hermetia illucens*, inoculation trial, organic side streams, rearing, *Staphylococcus aureus*

## Abstract

Black soldier fly larvae (BSFL; Hermetia illucens) are promising insects for the conversion of organic waste streams into valuable biomolecules. Such waste streams can contain foodborne pathogens. To assess this risk factor, this study evaluated the presence of Staphylococcus aureus in waste streams as a substrate ingredient for BSFL production as well as in the rearing process. First, the general microbiological quality and the occurrence of S. aureus were investigated for different waste streams. Staphylococcus aureus was abundantly present. Control of pH and water activity should avoid pathogens, which cannot grow in single-substrate ingredients, redeveloping when mixing streams for optimal substrate conditions for BSFL production. Next, it was investigated whether S. aureus present in the substrate was ingested and/or eradicated by BSFL. In inoculation trials, with S. aureus added to chicken feed as the substrate at 3 or 7 log CFU/g, the larvae showed a reducing effect on S. aureus. After 6 days, S. aureus counts were below the detection limit (2.0 log CFU/g) in all larvae samples and decreased in the substrate to <2.0 and <3.1 log CFU/g for inoculation levels of 3 and 7 log CFU/g, respectively. While this is promising, it is still recommended to monitor and control this pathogen in BSFL rearing. Intriguingly, screening of antimicrobial activity of dominant microorganisms associated with BSFL showed a clear activity of *Trichosporon* isolates against S. aureus. Future research should explore whether *Trichosporon*, which is frequently observed in BSFL, plays a role in controlling specific microorganisms, such as S. aureus.

**IMPORTANCE** Given the increasing need for (more sustainable) methods to upcycle organic waste streams, the interest to rear insects, like black soldier fly larvae (BSFL), on such streams is increasing. This study reveals that S. aureus is abundantly present in such waste streams, which might be a point of attention for insect producers. At the same time, it reveals that when S. aureus was inoculated in chicken feed as the substrate, it was not detected in the larvae and was reduced in the substrate after 6 days. Future inoculation trials should investigate whether this reduction is substrate dependent or not. Toward the future, the role of the BSFL microbiota in controlling intestinal bacterial community homeostasis should be explored, because one of the dominant microorganisms associated with BSFL, *Trichosporon* spp., showed clear activity against S. aureus.

## INTRODUCTION

Black soldier fly larvae (BSFL; *Hermetia illucens* L., Diptera: Stratiomyidae) are known to be highly efficient in the biodegradation of organic waste (1, 2). As they convert organic residues into valuable biomolecules, such as proteins, lipids, and chitin, these industrially reared insects can contribute to a more circular economy (3). Because BSFL do not have specific dietary restrictions (4), they are able to feed on a broad variety of organic waste streams, such as animal manure, fruit and vegetable waste, and food waste and municipal waste (1, 5–7). Despite the great potential of mass-reared insects, such as BSFL, to contribute to an improved valorization of organic waste streams, some restrictions are imposed by the European legislation. Because insects produced at industrial scale are considered to be farm animals, feeding substrates such as manure, catering waste, or leftovers containing meat or fish, is currently not allowed according to Regulation (EC) No. 1069/2009 (8, 9). Because organic waste streams (also the authorized ones) can obviously contain a broad range of (harmful) microorganisms, it is important to obtain a general overview of the microbial load and composition.

Recently, the three main bacterial pathogens associated with insects produced for food and feed (also termed “edible insects”) were identified to be Staphylococcus aureus, pathogenic *Clostridium* spp. (such as the species C. perfringens and C. botulinum), and pathogenic species of the Bacillus cereus group (10). The species S. aureus is a Gram-positive, nonsporulating, toxin-producing bacterium, which belongs to the normal flora of the human skin and mucosae as a commensal bacterium, but it also occurs as a human pathogen (11, 12). Indeed, S. aureus colonizes up to 30% of the human population (12, 13) and can cause a broad variety of diseases, ranging from superficial skin infections to more severe diseases, such as toxic shock syndrome or meningitis (14, 15). Treatment of these infections has become more and more challenging due to the emergence of methicillin-resistant S. aureus (MRSA) strains, which are resistant to all applicable beta-lactam antibiotics (13). Next to a human health problem, S. aureus (including MRSA) is also a problem for animal welfare because it can colonize a broad range of animals, including farm animals, such as pigs, cattle, and poultry (14). Moreover, the prevalence of S. aureus is dependent on the host species and varies from 14% to 35% in cows and heifers, up to 29% in sheep and 42% in pigs, to even 90% in chickens (16, 17). When infecting farm animals, S. aureus also interferes with economic yield. Staphylococcal mastitis in ruminants, for example, is a real problem in the dairy industry and can even result in losses of up to €300 per cow per year (14, 16). For insects, most of the entomopathogenic bacteria belong to a number of bacterial families, such as Bacillaceae, Enterobacteriaceae, Micrococcaceae, Pseudomonadaceae, and Streptococcaceae, while S. aureus is currently not listed as an entomopathogen (18). However, insects can function as a carrier of pathogens and transmit the pathogen to other hosts by contact transfer (mostly for flying insects) or by ingestion (for edible insects) (10, 19, 20).

A few authors studied the occurrence of several pathogens in BSFL and reported the presence of S. aureus in the larvae and/or residue after rearing (21–23). Nevertheless, information on the occurrence of S. aureus in organic waste streams used as rearing substrates for BSFL is lacking. Moreover, it is not yet described whether BSFL would accumulate or rather eradicate S. aureus cells when the pathogen would enter the larvae via ingestion with the substrate.

To narrow this knowledge gap, the risk of S. aureus when present in substrates for BSFL rearing was evaluated. To this end, the presence of S. aureus was investigated in a range of potential substrate ingredients. At the same time, other microbiological parameters were included to obtain a general overview of the microbiological quality of the streams. Further, the dynamics of S. aureus present in the substrate and the larvae during rearing were assessed by inoculating known cell densities of the pathogen in the substrate administered to the larvae. Because a suppression of S. aureus was noticed in the inoculation trials, antimicrobial activity of dominant microorganisms of BSFL against S. aureus was evaluated to explore whether members of the larval microbiota could play a role in this reduction.

## RESULTS

### Intrinsic parameters of the waste streams.

In dialogue with BSFL producers, 10 different waste streams were selected. The streams originated from agriculture, the food or feed industry, or from food waste processors, and they were judged to have the potential to be used as a substrate (ingredient) for BSFL rearing. In that way, the residual streams can be upcycled and contribute to a more circular economy and sustainable waste management. Selected waste streams were chicken litter, overproduced vegetables from an auction, corn meal, grain mix, apple pulp, fruit puree, catering/supermarket/industrial food waste, household food waste, strawberry leaves, and tomato leaves. Strawberry and tomato leaves were sampled once, whereas the other waste streams were sampled at two different times.

As intrinsic parameters, the pH, the water activity (*a*_w_), and the moisture content of each waste stream were determined. The results of these intrinsic parameters are shown in Table 1. The pH was measured for samples obtained during two seasons or periods, while the water activity and moisture content were only measured in the second sampling period. All waste streams showed a pH below 7.00 except chicken litter, which had a higher pH of 8.40 to 8.86. The lowest pH for sampling period 1 was observed for apple pulp and fruit puree, while in period 2, only fruit puree showed a pH below 4.00. In addition, the two food waste samples (catering/supermarket/industrial food waste and household food waste) showed a low pH below 4.60. Whereas for tomato leaves a comparable average pH of 4.32 was determined, strawberry leaves showed a significantly higher pH of 5.43 (*P* < 0.001). Overproduced vegetables from the auction were characterized with a pH of around 5.00, while the dry waste streams corn meal and grain mix were characterized by a pH between 5.90 and 6.50. When comparing sampling period 1 and 2, only the pH of fruit puree and catering/supermarket/industrial food waste did not differ significantly between periods (*P* > 0.999 and *P* = 0.077, respectively).

**TABLE 1:** Intrinsic parameters of the waste streams sampled in periods 1 and 2

	Intrinsic parameters^*a*^^,^^*b*^			
Waste stream	pH (−)		Water activity (*a*_w_) (−)	Moisture content (%)
	Period 1	Period 2	Period 2	Period 2
Chicken manure	8.86 ± 0.14 B,h	8.40 ± 0.13 A,f	0.98 ± 0.01 c	37.6 ± 0.9 b
Vegetable auction overproduction	5.07 ± 0.02 B,e	4.85 ± 0.02 A,c	0.98 ± 0.00 c	88.5 ± 0.3 f
Corn meal	6.19 ± 0.02 B,g	5.95 ± 0.01 A,d	0.73 ± 0.00 b	13.4 ± 0.7 a
Grain mix	5.91 ± 0.06 A,g	6.43 ± 0.05 B,e	0.69 ± 0.00 a	13.9 ± 1.1 a
Apple pulp	3.76 ± 0.01 A,a	4.33 ± 0.05 B,b	0.98 ± 0.00 c	71.2 ± 0.5 c
Fruit puree	3.89 ± 0.02 A,a,b	3.87 ± 0.02 A,a	0.98 ± 0.00 c	94.9 ± 0.1 g
Catering/supermarket/industrial food waste	4.17 ± 0.01 A,b,c	4.30 ± 0.05 A,b	0.97 ± 0.00 c	77.6 ± 0.8 e
Household food waste	4.51 ± 0.02 B,d	4.36 ± 0.05 A,b	0.97 ± 0.00 c	75.3 ± 0.8 d
Strawberry leaves^*c*^	5.43 ± 0.29 f	ND	ND	ND
Tomato leaves^*c*^	4.32 ± 0.06 c,d	ND	ND	ND

a Results are presented as the mean of three replicates ± standard deviation. Mean pH from different sampling periods of the same waste stream that share a capital letter (A, B) within the same row are not statistically different (*P* ≥ 0.05). Mean intrinsic parameters for waste streams that share a lowercase letter (a to h) within the same column are not statistically different (*P* ≥ 0.05).

b ND, not determined.

c For strawberry leaves and tomato leaves, only data for sampling period 1 were obtained.

Concerning water activity, a high *a*_w_ of 0.97 to 0.98 was measured for most of the waste streams in period 2 (no data were obtained for period 1). In contrast, a lower *a*_w_ was observed for corn meal and grain mix. Similarly, the moisture content in period 2 (no data were obtained for period 1) showed the lowest, yet comparable, values for corn meal and grain mix (13.4% and 13.9%, respectively; no significant difference, *P* = 0.986). While for chicken litter a high *a*_w_ of 0.98 was obtained, a relatively low average moisture content of 37.6% was found. All other waste streams showed a moisture content above 70.0%, with the two food waste streams being similar (yet statistically different; *P* = 0.017) and fruit puree showing the highest moisture content.

### General microbiological quality of the waste streams.

The microbiological counts obtained for the waste streams are shown in Table 2. For strawberry and tomato leaves, only results for period 1 were obtained. Not surprisingly, the highest values for the total viable count were observed for chicken litter, and they even exceeded 10.0 log CFU/g. The total viable count for all other waste streams, except for corn meal, ranged between 6.3 and 9.4 log CFU/g. The lowest values were observed for corn meal, with an average total viable count of 5.2 log CFU/g for both sampling periods. For the Enterobacteriaceae, average counts were again the highest for chicken litter (8.3 log CFU/g) in period 1, followed by strawberry leaves (7.9 log CFU/g). Other waste streams showed variable counts, ranging from very low counts (2.2 log CFU/g) to higher counts (6.9 log CFU/g). For some of the waste streams (apple pulp in period 1 and fruit puree for both periods), the Enterobacteriaceae were even below the detection limit of 1.0 log CFU/g. Another bacterial group, the lactic acid bacteria (LAB), was observed in all waste streams. The dry waste streams (corn meal and grain mix), overproduced vegetables from the auction, and apple pulp (period 2) showed average values between 3.5 and 6.2 log CFU/g, whereas the other waste streams were characterized by higher values for LAB, all ranging between 8.2 and 9.3 log CFU/g. For apple pulp, fruit puree, catering/supermarket/industrial food waste, household food waste, and tomato leaves, LAB also appeared to be the most dominant microbial group with values close to the total viable count. Large differences were also observed for the aerobic bacterial endospores and yeasts and molds, ranging from 2.2 to 7.2 log CFU/g and from 3.3 to 8.1 log CFU/g, respectively. Lower values were obtained for sulfite-reducing clostridia with the highest average counts for household food waste (3.8 log CFU/g). All other observed waste streams showed average counts between 1.5 and 2.8 log CFU/g, with the exception of the low pH streams apple pulp and fruit puree for which sulfite-reducing clostridia were below the detection limit of 1.0 log CFU/g. Because sulfite-reducing clostridia were only measured in sampling period 2, no results for this parameter are available for strawberry leaves and tomato leaves. Further, the food pathogen Salmonella was observed to be present in small amounts in chicken litter and grain mix but only in one replicate in period 1, resulting in an average count of less than 3.2 and 2.2 log CFU/g, respectively. For all other waste streams, counts for Salmonella were below the detection limit of 2.0 log CFU/g.

**TABLE 2:** Microbial counts obtained for the waste streams sampled in periods 1 and 2

Waste stream	Sampling period	Microbial counts (log CFU/g)^*a*^^,^^*b*^						
		Total viable counts	Enterobacteriaceae	Lactic acid bacteria	Aerobic bacterial endospores	Yeasts and molds	Sulfite-reducing clostridia	Salmonella spp.
Chicken litter	1	10.6 ± 0.1 B	8.3 ± 0.3 B	8.5 ± 0.2 A	5.4 ± 0.2 A	6.1 ± 0.6 A	ND	<3.2 ± 2.1 A
	2	10.2 ± 0.2 A	5.3 ± 0.2 A	8.7 ± 0.2 A	6.3 ± 0.4 B	5.6 ± 0.1 A	2.8 ± 0.4	<2.0 ± 0.0 A
Vegetable overproduction auction	1	7.0 ± 0.1 A	2.7 ± 0.3 A	3.5 ± 0.4 A	2.7 ± 0.1 A	3.3 ± 0.0 A	ND	<2.0 ± 0.0 A
	2	8.0 ± 0.1 B	2.2 ± 0.1 A	5.2 ± 0.0 B	5.1 ± 0.0 B	5.1 ± 0.0 B	2.5 ± 0.1	<2.0 ± 0.0 A
Corn meal	1	5.2 ± 0.6 A	4.3 ± 0.9 A	4.3 ± 0.1 B	3.8 ± 0.2 B	3.7 ± 0.3 A	ND	<2.0 ± 0.0 A
	2	5.2 ± 0.1 A	4.5 ± 0.3 A	3.8 ± 0.2 A	2.4 ± 0.0 A	4.1 ± 0.1 A	1.5 ± 0.1	<2.0 ± 0.0 A
Grain mix	1	7.7 ± 0.0 A	5.0 ± 0.2 A	5.9 ± 0.1 B	5.8 ± 0.5 B	5.5 ± 0.1 A	ND	<2.2 ± 0.3 A
	2	8.8 ± 0.0 B	6.8 ± 0.2 B	5.5 ± 0.1 A	4.2 ± 0.4 A	5.4 ± 0.4 A	2.9 ± 0.2	<2.0 ± 0.0 A
Apple pulp	1	9.0 ± 0.0 B	<1.0 ± 0.0 A	9.1 ± 0.0 B	4.7 ± 0.4 B	5.7 ± 0.1 B	ND	<2.0 ± 0.0 A
	2	6.3 ± 0.2 A	2.3 ± 0.2 B	6.2 ± 0.1 A	2.2 ± 0.3 A	4.7 ± 0.3 A	<1.0 ± 0.0	<2.0 ± 0.0 A
Fruit puree	1	8.6 ± 0.6 A	<1.0 ± 0.0 A	8.8 ± 0.1 A	3.5 ± 0.3 A	7.1 ± 0.1 A	ND	<2.0 ± 0.0 A
	2	7.9 ± 0.1 A	<1.0 ± 0.0 A	8.8 ± 0.0 A	3.0 ± 0.1 A	8.1 ± 0.0 A	<1.0 ± 0.0	<2.0 ± 0.0 A
Catering/supermarket/industrial food waste	1	8.3 ± 0.0 A	3.4 ± 0.1 A	8.9 ± 0.0 B	6.0 ± 0.1 B	6.4 ± 0.2 A	ND	<2.0 ± 0.0 A
	2	8.4 ± 0.0 A	5.9 ± 0.1 B	8.5 ± 0.0 A	5.8 ± 0.0 A	7.0 ± 0.2 B	2.8 ± 0.0	<2.0 ± 0.0 A
Household food waste	1	8.1 ± 0.2 A	3.8 ± 0.1 A	8.5 ± 0.1 A	7.2 ± 0.0 B	5.8 ± 0.1 A	ND	<2.0 ± 0.0 A
	2	9.2 ± 0.1 B	6.9 ± 0.1 B	9.3 ± 0.0 B	6.3 ± 0.0 A	7.2 ± 0.1 B	3.8 ± 0.1	<2.0 ± 0.0 A
Strawberry leaves^*c*^	1	9.4 ± 0.5	7.9 ± 0.5	8.2 ± 0.2	4.6 ± 0.1	6.6 ± 1.0	ND	<2.0 ± 0.0
Tomato leaves^*c*^	1	8.3 ± 0.0	2.8 ± 2.0	8.3 ± 0.0	3.6 ± 0.3	5.1 ± 0.4	ND	<2.0 ± 0.0

a Results are presented as the mean of three replicates ± standard deviation. Mean plate counts from different periods of the same waste stream that share a letter (A, B) are not statistically different (*P* ≥ 0.05).

b ND, not determined.

c For strawberry leaves and tomato leaves, only data for sampling period 1 were obtained.

### Occurrence of Staphylococcus aureus in the waste streams.

**FIG 1:**
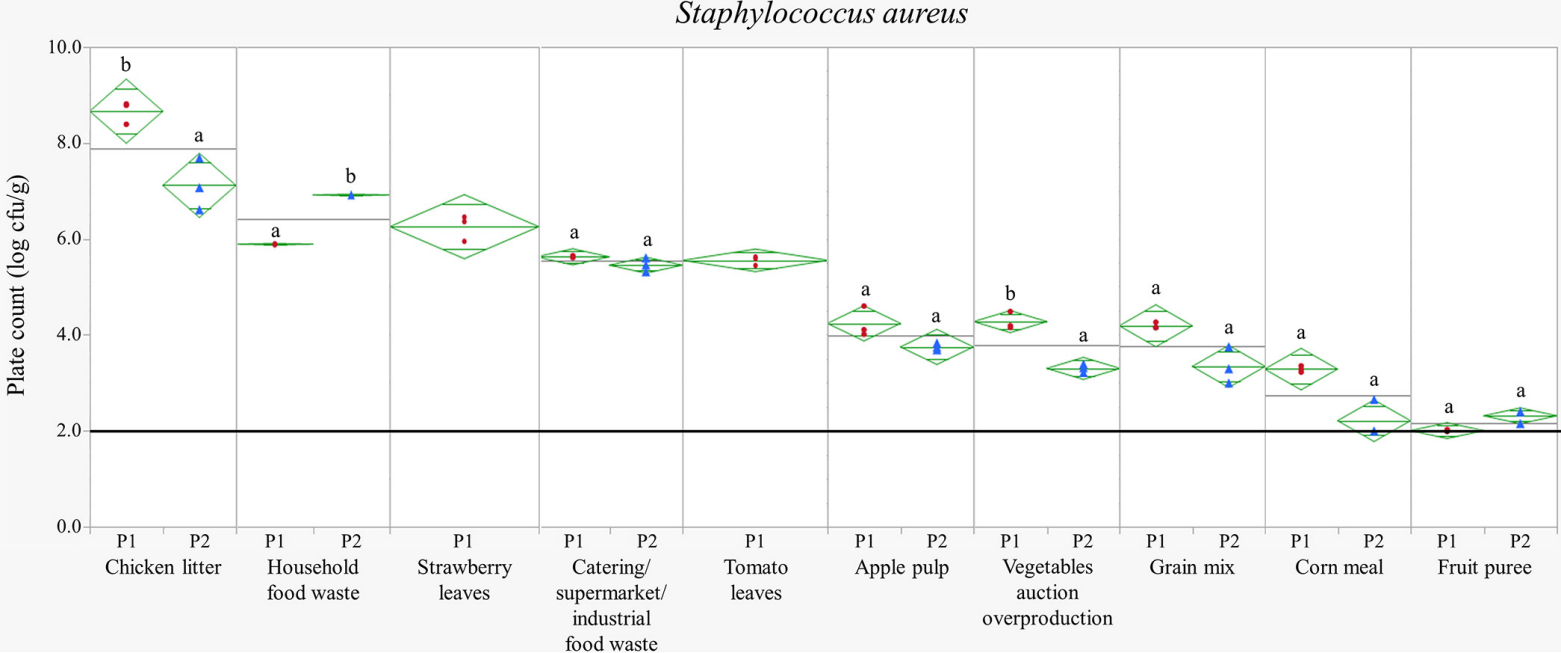
Mean diamonds plots representing the Staphylococcus aureus counts in log CFU/g of the waste streams sampled in periods 1 and 2. The symbols ● and ▴ represent the replicates in period 1 (P1; June to September) and period 2 (P2; January to February in the year after sampling period 1), respectively. The top and bottom of each diamond represent the 100% confidence interval for each period, and the horizontal line in the middle of each diamond represents the mean for each period. The 95% confidence marks are indicated as lines above and below the mean. The horizontal black lines represent the mean plate count of all replicates of both sampling periods for each waste stream. The thick horizontal black line represents the detection limit (2.0 log CFU/g). Mean plate counts from different periods of the same waste stream that share a letter (a and b) are not statistically different (*P* ≥ 0.05). For strawberry leaves and tomato leaves, only period 1 data are available.

Special attention was given to the presence of S. aureus in the waste streams. Figure 1 shows the counts for the pathogen. S. aureus was observed in all replicates of all waste streams, with the exception of fruit puree. For the latter stream, values were below the detection limit of 2.0 log CFU/g in period 1, while S. aureus was found in all three replicates in period 2 but only at an average count of 2.3 log CFU/g. The highest values were measured for chicken litter, ranging from 7.1 to 8.7 log CFU/g. Also catering/supermarket/industrial food waste showed high values of 5.5 to 5.6 log CFU/g in both sampling periods as well as household food waste, which showed a significantly lower average value in period 1 (5.9 log CFU/g) than in period 2 (6.9 log CFU/g; *P* < 0.001). A similar level of S. aureus was observed for strawberry leaves (5.6 log CFU/g) and tomato leaves (6.3 log CFU/g). Somewhat lower levels were measured for apple pulp, overproduced vegetables from the auction, and grain mix, all ranging from 3.3 to 4.3 log CFU/g. For corn meal, low average values were also detected and were slightly (but not significantly) higher in period 1 (3.3 log CFU/g) than in period 2 (2.2 log CFU/g; *P* = 0.081).

### Dynamics of Staphylococcus aureus during rearing of BSFL on chicken feed.

Next to the occurrence of the pathogen in potential substrate ingredients, it was also investigated how the pathogen behaves during rearing when it is effectively present in the substrate for the larvae. The results of the total viable counts and the S. aureus counts recorded during the inoculation trials are presented in Table 3. A significant increase in the total viable counts was noticed during the experiments for all substrate samples (irrespective of the presence of larvae or inoculation with S. aureus), with values between 10.1 and 11.1 log CFU/g at day 6. At day 0, the substrate inoculated with S. aureus resulted in higher total viable counts than the control replicates, which was evidently due to the inoculation. For the larval samples, the total viable counts also increased significantly over the course of the experiments, with already high initial values at day 0 (ranging between 7.3 and 8.6 log CFU/g). The total viable counts of the larvae at day 6 were lower than those of the substrate for all conditions.

**TABLE 3:** Total aerobic viable counts and Staphylococcus aureus counts from larvae and substrate (chicken feed) samples

Experimental condition^*a*^	Sample	Target S. aureus contamination level in chicken feed (log CFU/g)	Total viable count (log CFU/g)^*b*^			S. aureus count (log CFU/g)^*b*^		
			Day 0	Day 2	Day 6	Day 0	Day 2	Day 6
CF	Substrate	Control	4.0 ± 0.1 A	9.8 ± 0.2 B	10.2 ± 0.1 C	<2.0^†^ A	<2.0^†^ A	<2.0^†^ A
CF + L	Substrate	Control	3.7 ± 0.1 A	10.1 ± 0.1 B	10.6 ± 0.2 C	<2.0^†^ A	<2.0^†^ A	<2.0^†^ A
CF + L	Larvae	Control	8.6 A	9.6 ± 0.3 B	9.6 ± 0.2 B	<2.0^†^ A	<2.0^†^ A	<2.0^†^ A
CF + P	Substrate	3	4.6 ± 0.4 A	9.6 ± 0.1 B	10.1 ± 0.1 C	3.8 ± 0.1 A	3.7 ± 0.4 A	<3.0 ± 0.1° B
CF + P + L	Substrate	3	5.0 ± 0.2 A	10.0 ± 0.1 B	10.6 ± 0.1 C	3.5 ± 0.2 A	<2.1 ± 0.2° B	<2.0 ± 0.0° B
CF + P + L	Larvae	3	8.3 A	9.1 ± 0.1 B	9.1 ± 0.2 B	<2.0^†^ A	<2.0^†^ A	<2.0^†^ A
CF + P	Substrate	7	7.3 ± 0.2 A	9.3 ± 0.1 B	10.3 ± 0.3 C	7.2 ± 0.2 A,B	8.0 ± 0.2 B	<6.2 ± 0.8° A
CF + P + L	Substrate	7	7.3 ± 0.3 A	10.1 ± 0.1 B	11.1 ± 0.5 C	7.3 ± 0.1 A	4.7 ± 0.8° B	<3.1 ± 1.2° B
CF + P + L	Larvae	7	7.3 ± 0.3 A	9.4 ± 0.8 B	9.2 ± 0.2 B	<2.0^†^ A	<2.7 ± 0.6° A	<2.0^†^ A

a CF, chicken feed; CF + P, chicken feed with pathogen; CF + L, chicken feed with larvae; CF + P + L, chicken feed with pathogen and larvae.

b Results are presented as the mean ± standard deviation of six replicates per condition. Average values for total viable counts and S. aureus counts within each row that share a letter (A, B, C) did not significantly (*P* ≥ 0.05) increase or decrease between sampling days. The notation “<2.0^†^” indicates that Staphylococcus aureus was below the detection limit (2.0 log CFU/g) in every sample. The ° notation with a value higher than 2.0 log CFU/g indicates that the S. aureus count was below the detection limit (varying between 2.0 log CFU/g and 5.0 log CFU/g) in at least one sample but not all samples.

The counts for S. aureus for both the substrate as well as the larvae of the control replicates were below the detection limit for each sampling day. This shows that in inoculated samples, the S. aureus counts resulted only from the inoculation. The aim of the inoculation trial was to achieve an inoculation level of 3 or 7 log CFU of S. aureus per gram of substrate. The counts for S. aureus obtained at day 0 were indeed close to the target inoculation level, being 3.5 to 3.8 and 7.2 to 7.3 log CFU/g on average, respectively. When the chicken feed was inoculated with a target level of 3 log CFU/g (and in the absence of larvae), the S. aureus counts did not significantly change during the first 2 days of the experiment, indicating that the pathogen can survive, at least for a short period of time, in the substrate when it is kept under rearing conditions. However, at day 6, the counts were significantly lower (i.e., they were ≤3.0 log CFU/g, with five samples under the detection limit of 3.0 log CFU/g). When larvae were present, the S. aureus counts of the substrate decreased faster, where at day 2, four samples were under the detection limit of 2.0 log CFU/g. Interestingly, the S. aureus counts of the larvae were below the detection limit of 2.0 log CFU/g for all sampling days. Chicken feed inoculated at the high inoculation level of 7 log CFU/g resulted in higher S. aureus counts over the duration of the trial. At day 6, the S. aureus count reached an average of ≤6.2 log CFU/g, with one sample under the detection limit of 5.0 log CFU/g. Addition of larvae to the inoculated chicken feed resulted in lower S. aureus counts of the substrate than the counts of substrate without larvae. A significant decrease from 7.3 at day 0 to ≤3.1 log CFU/g at day 6 was observed, with one sample at day 6 under the detection limit of 2.0 log CFU/g. The pathogen counts of the larvae were under the detection limit of 2.0 log CFU/g for all samples at day 6. In addition to monitoring the pathogen during rearing, larval growth was monitored during the inoculation trials. The presence of S. aureus did not affect the growth of the larvae (data not shown).

### Exploring the role of abundant microorganisms in BSFL in the reduction of Staphylococcus aureus counts.

**FIG 2:**
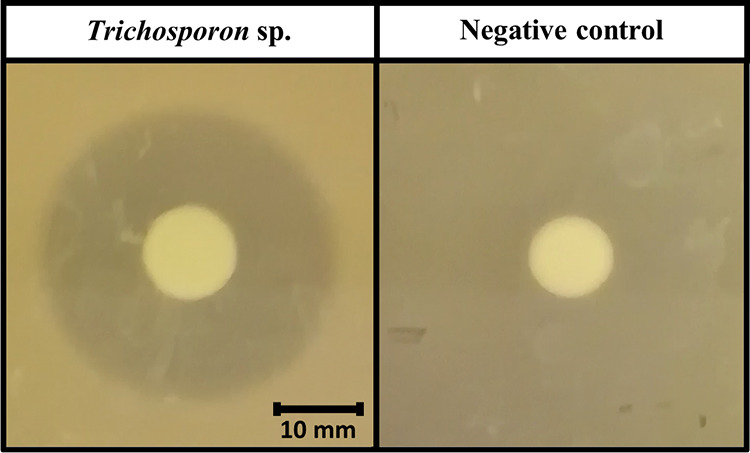
Inhibition zone illustrating the antimicrobial activity of a *Trichosporon* species isolate against Staphylococcus aureus (left) and a negative control (isolate without activity against S. aureus) (right).

Based on reports in the literature on the gut microorganisms in insects interfering with the growth of other microorganisms, the existing microbiota was also probed for activity against S. aureus. Therefore, an antimicrobial screening against S. aureus was performed on a collection of dominant microorganisms previously obtained in reference 24. From the 178 tested isolates, a majority of the 103 isolates (58%) showed signs of activity against S. aureus (i.e., an inhibition zone with a measurable radius). However, only the six fungal isolates, all identified as *Trichosporon* spp., exhibited strong antistaphylococcal activity. Strong activity was defined as either a large inhibition zone (radius of ≥10 mm) and a moderate clarity (≥50% inhibition) or a moderately sized inhibition zone (radius of ≥5 mm) and a high clarity (near complete inhibition) (25). All *Trichosporon* spp. showed an inhibition zone with a radius between 16 and 19 mm and moderate clarity. Figure 2 shows the inhibition zone of one of the *Trichosporon* spp. against S. aureus compared with the absence of an inhibition zone for an isolate without activity against S. aureus (negative control).

## DISCUSSION

### Microbiological quality of waste streams as substrate ingredient for BSFL rearing.

In this study, the overall microbiological quality of 10 waste streams that can potentially be used as substrate ingredients for BSFL rearing was investigated, with special focus on the occurrence of the food pathogen S. aureus. Based on Table 2, a first general finding is that there is broad variation in the total microbial load and in the microbial groups between the different waste streams. The total aerobic counts ranged from 5.2 log CFU/g for corn meal to values above 10.0 log CFU/g for chicken litter. Similar high values for chicken manure were also reported by other authors (26–28). Previous studies demonstrated that BSFL can grow well on chicken litter (29, 30). At the same time, the larvae can change the microbial community of the manure (31) and even reduce specific food pathogens, such as Escherichia coli, Salmonella, or S. aureus, when growing on manure (32, 33). Rearing BSFL on manure is currently not allowed in Europe, however, as defined in Regulation (EC) No. 1069/2009 (9).

**FIG 3:**
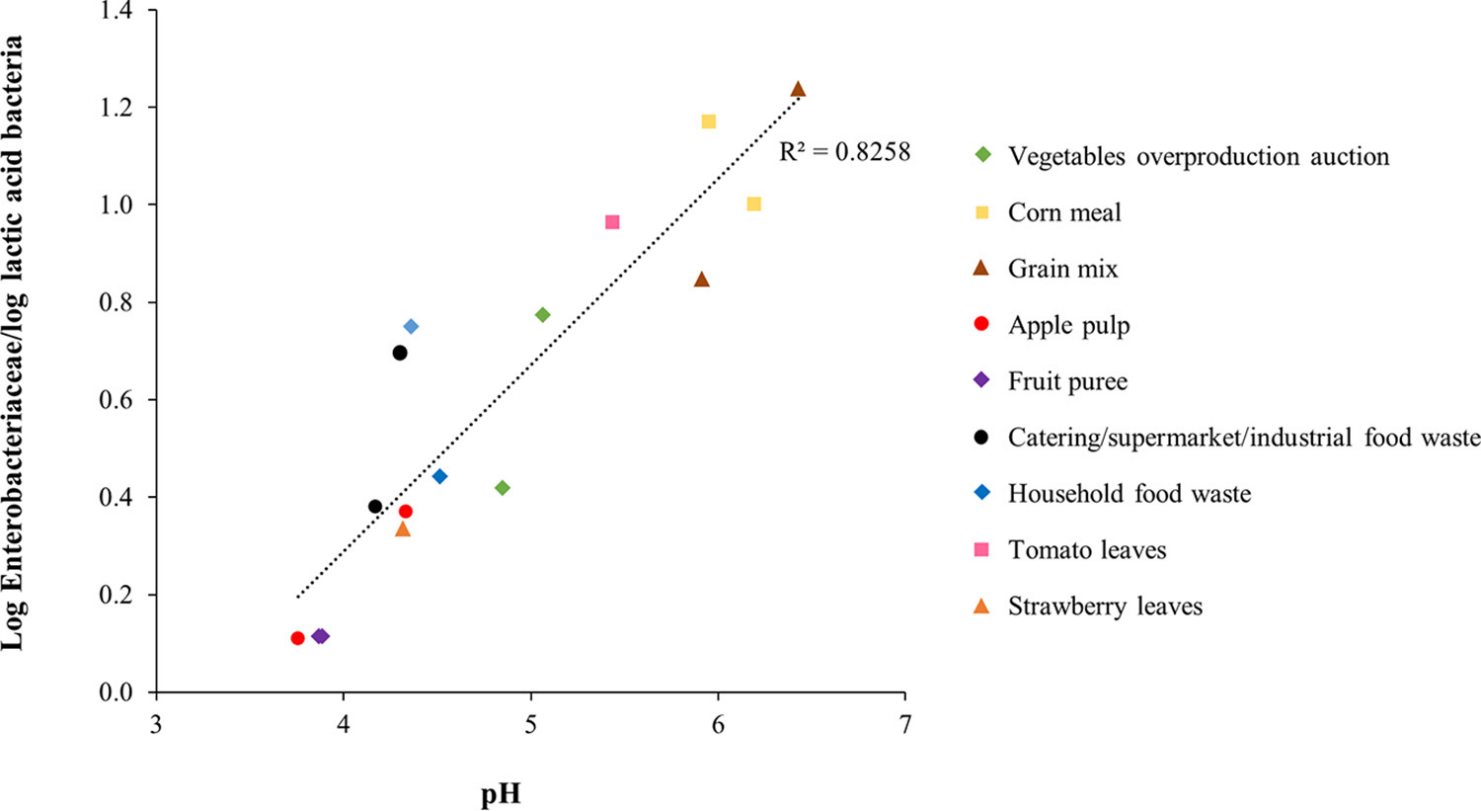
Relation between the Enterobacteriaceae/lactic acid bacteria ratio and the pH for all waste streams with a pH below 7.0.

A second observation is that the pH of the streams affects the amount of Enterobacteriaceae and LAB present. The relation between the Enterobacteriaceae/LAB ratio and the pH is visualized in Fig. 3 for all waste streams with a pH below 7.0 (only chicken litter was excluded). A linear relationship is observed, which shows that a lower pH coincides with a lower dominance of Enterobacteriaceae and a higher dominance of LAB, illustrated by a decreasing log Enterobacteriaceae/log LAB ratio. For those waste streams with a pH above 4.6, the Enterobacteriaceae and LAB showed comparable counts, except for overproduced vegetables in period 2. In waste streams with a pH between 4.0 and 4.6, both microbial groups were also detected but with a strong dominance of the LAB. LAB showed values that were 2.5 to 5 log cycles higher than values for the Enterobacteriaceae. This is illustrated by values below 1.0 for the log Enterobacteriaceae/log LAB ratio in Fig. 3. For waste streams with a pH below 4.0, the acidic environment likely had a bacteriostatic effect on the Enterobacteriaceae. In contrast, LAB were able to thrive well under these conditions, as illustrated by high counts of approximately 9 log CFU/g. Likely, the LAB produced lactic acid themselves and in this way also decreased the pH (34–37). Because several studies (4, 38) reported that BSFL are able to grow in acidic environments, and other authors (39) stated that most pathogenic bacteria are unable to grow below a pH of 4.0, the use of low-pH waste streams (e.g., fermented streams) can be an interesting strategy to limit growth of food pathogens in the rearing substrates of BSFL. However, larval metabolic activity will inevitably lead to an increase in the pH of the substrate during rearing (4, 38). This pH increase can result in redevelopment and growth of dormant pathogen cells and endospores that remained present in the substrate.

A third finding that may not be neglected is the presence of aerobic endospores in the waste streams. Table 2 shows that some streams are characterized with average counts above 5.0 log CFU/g, including chicken litter, overproduced vegetables from the auction (period 2), grain mix (period 1), catering/supermarket/industrial food waste, and household food waste. If these bacterial endospores present in the substrate are ingested and remain in the larvae during the rearing process, it is possible that they are not completely eliminated by a postharvest heat processing step of the insects (40). Because this group of aerobic endospores can include pathogenic bacteria, such as species from the B. cereus group, it is recommended to limit the use of substrates that contain substantial amounts of endospores for insect rearing (41). For the same reason, anaerobic endospores, such as those produced by *Clostridium* species, including the pathogenic species C. perfringens and C. botulinum, should preferably not be present in rearing substrates. Adequate quality control is of high importance in this context (41).

Furthermore, attention should be paid to the presence of yeasts and molds in the waste streams because the composition of the fungal community of BSFL is impacted mainly by the feeding substrate (42). The use of various waste streams in substrates, such as vegetable waste, increases the fungal diversity in comparison with chicken feed (42). High counts for yeasts and molds were observed in this study for low-pH waste streams, especially for fruit puree (Table 2). Indeed, at pH values above 4.0, bacteria dominate the microbial community, while at pH values below 4.0, yeasts and molds become more prevalent (43). The fact that certain fungi can produce mycotoxins may cause safety problems for BSFL rearing. The fate of mycotoxins in substrates for BSFL rearing has already been a topic of research, for instance in references 44–47, and is still under investigation.

Sulfite-reducing clostridia were also considered in assessing the microbial profile of the possible substrate ingredients, as food pathogens from the spore-forming genus *Clostridium* belong to the most pertinent risks of edible insects in terms of microbiological food safety (10). Of all waste streams studied for sulfite-reducing clostridia, only in apple pulp and fruit puree was this bacterial group not detected. The low pH of these waste streams could explain the inability of clostridia to grow. This inhibitory effect of acidic environments was also demonstrated for C. botulinum (48), C. perfringens (49), and C. sporogenes (50), with a minimal pH of 4.5 required for the growth of vegetative *Clostridium* cells; however, the effect of pH was strain and time dependent. Although sulfite-reducing clostridia were detected at (mostly) low values in other waste streams (Table 2), *Clostridium* cells do not necessarily survive in or colonize the gut when ingested by BSFL. Previous research (51) demonstrated that C. perfringens cells were not present in the larvae even if they were in the substrate. As mentioned before, the production of endospores and/or toxins should also be taken into account when assessing the safety of BSFL-rearing substrates contaminated with sulfite-reducing clostridia.

Finally, the food pathogen Salmonella was barely detected in the organic waste streams. Hence, Salmonella appeared to be not highly relevant regarding the microbiological safety of the considered substrates for BSFL rearing. This is a favorable outcome since the authors of a previous study (52) recommended using substrate ingredients free from Salmonella for BSFL rearing because eradication of Salmonella by the larvae could not be demonstrated.

Aside from the variation between different waste streams, variations in microbial counts between the two sampling periods of the same waste stream can also be observed in Table 2. Residual streams from industrial production involving several processing steps revealed a stable microbial composition with little variation between time points and batches. Waste streams such as food waste streams, however, showed much more variation in microbial counts between the two sampling periods. It can be assumed that the composition of the streams is a key factor in this variation because side streams from industrial processes likely have a more uniform composition than food waste samples, for example, which may include several food sources. Also, more stringent intrinsic factors, such as a low pH or water activity, can possibly contribute to a more consistent microbial community. As to microbial subgroups, the largest variation between the two periods was observed for the Enterobacteriaceae and aerobic endospores.

Because organic waste streams are often characterized by a high microbial load, and the presence of food pathogens is not unusual, rearing of BSFL on organic residues will inevitably lead to more food safety monitoring and control. This is important for legislators, as currently, according to EU legislation (EC No. 767/2009 and EC No. 1069/2009), some of the waste streams considered in this study, for example chicken manure and household food waste, are not yet allowed as feed material for BSFL in the European Union (8, 9, 53). However, the fact that waste streams can contain various food pathogens does not necessarily mean that the larvae are contaminated as well. To set up appropriate risk assessments, more information on the transfer of food pathogens from the substrate to the larvae, as implemented in this study for S. aureus, is required. At the same time, downstream processing steps will also impact the final microbiological safety on insect products.

### Occurrence of Staphylococcus aureus in waste streams as substrate ingredients.

According to Fig. 1, S. aureus seemed to be ubiquitous in the studied waste streams. Not surprisingly, the highest plate counts were noted for chicken litter. Similar values were also reported in other studies, in which Staphylococcus counts of 5.8 to 7.3 log CFU/g were determined for laying hen excreta (26) and even higher values of 11.1 to 13.3 log CFU/g for litter from laying hens and broilers (28). Inhibition of growth of S. aureus can possibly be caused by a low pH (as illustrated in fruit puree) because S. aureus is characterized by a pH range for growth of 4.0 to 10.0 (11, 54, 55). Moreover, other authors (56) detected S. aureus in only 5% of the 32 studied fruit waste samples obtained from supermarkets or shops, with average values of 2.0 to 2.6 log CFU/g. Unlike fruit puree, apple pulp was also characterized by a pH below 4.0 but showed higher values for S. aureus. This can be explained by the fact that microbial growth at a specific pH is also influenced by other environmental conditions, such as water activity and temperature and acid causing the low pH (57). Furthermore, for the waste streams with a pH between 4.0 and 4.6, S. aureus was observed in larger amounts (above 5.0 log CFU/g). Also, other studies (56, 58) reported the presence of S. aureus in food waste. One of these studies (56) revealed average S. aureus counts of 5.9 log CFU/g for restaurant food waste and 4.5 log CFU/g for household food waste, which is in line with the values obtained for food waste in this study.

Next to the pH, water activity of the matrix can influence the growth of S. aureus (59–61). The minimal water activity for growth of 0.86 for this pathogen (59–61) can, at least partially, explain the low counts for S. aureus in the two dry streams corn meal and grain mix, which are both characterized by a low water activity. Although chicken litter is also a relatively dry stream with a low moisture content, it still has a high *a*_w_ of 0.98. Hence, the high values of S. aureus (and also other microorganisms) for chicken litter illustrate the fact that water activity is crucial to control microbial growth and toxin production rather than moisture content (43). The inhibition of bacterial growth by low water activity, but also by a low pH, can create opportunities to preserve waste streams for BSFL rearing. Although a low water activity can slow down or inhibit bacterial growth, (pathogenic) bacteria can still survive and eventually redevelop when the water activity increases again (43). This must be taken into account when mixing dry waste streams with more wet streams to create more suitable substrate conditions for BSFL rearing. As reported in a previous study (62), larval development seemed to fail at a moisture content below 40%, while a moisture content of 70% leads to optimal larval growth. Hence, mixing of waste streams to obtain that moisture content could also lead to more suitable conditions for S. aureus and other pathogens to multiply.

Not only the growth and/or presence of S. aureus should be controlled, as the presence of staphylococcal food-poisoning toxins synthesized by the pathogen should not be neglected. These heat-stable enterotoxins can survive and remain active under conditions in which the bacterial cells are killed (63). Hence, it cannot be excluded that staphylococcal toxins are ingested by the larvae when present in the rearing substrate and enter the rest of the food chain via the processed larvae.

### Dynamics of Staphylococcus aureus during rearing of BSFL on chicken feed.

To find out how S. aureus behaves when it is present in the substrate, it was inoculated at known levels in chicken feed. This feed is a substrate that is more defined than the waste streams investigated in the first part of the study, and it is also being used in industrial rearing as starter feed for young larvae.

In preliminary tests (not described in this paper), a high abundance of background microbiota on the selective plates was observed, disturbing the specific monitoring of the inoculated strain. This resulted in the use of a kanamycin-resistant strain combined with the addition of the antibiotic in the medium as an additional selective mechanism. Nevertheless, a background microbiota (presumably molds) was still present despite this selective aid, leading to the addition of dichloran as a mold-spreading inhibitor. A second hurdle observed during preliminary tests was cross-contamination, as was observed in inoculation trials with Salmonella spp. as well (52). Here, cross-contamination was prevented by incubating the noninoculated and inoculated replicates in separate climate chambers. Airborne S. aureus bacteria and transmission in different large-scale environments, such as in health care facilities, animal breeding units, wastewater treatment plants, and so on, have been described (64). Therefore, it is likely that airborne transmission of S. aureus can also take place in industrial facilities for rearing BSFL or other insects.

Once the setup was optimized, no S. aureus was detected in any control replicate (both substrate as larvae; see Table 3). This suggests that only the added kanamycin-resistant S. aureus strain was monitored. Because incubation and detection of kanamycin-resistant S. aureus occurred on Vogel-Johnson agar (VJA) with the addition of kanamycin, naturally occurring S. aureus could not grow on these plates. It is thus important to note that this study did not examine whether naturally occurring S. aureus bacteria were present in the substrate or larvae; instead, it monitored the dynamics of the inoculated strain specifically.

For all conditions, the total viable count of the chicken feed significantly increased to values up to 11.1 log CFU/g regardless of the presence of larvae. Similar counts of chicken feed during BSFL rearing have been reported (23, 52). The high moisture content (circa 50%) and incubation temperature of 27°C are ideal for microbial growth. Interestingly, the presence of larvae did not affect the microbial load of the substrate in quantitative terms. High initial total viable counts for the larvae, in this study between 7.3 and 8.6 log CFU/g, have been noticed in other studies as well (23, 52). The total viable counts of the larvae at day 6 were for all conditions lower than those of the substrate. This suggests that some sort of control mechanism restricting the microbial load in the larvae exists, as also suggested by previous studies (52).

Pathogen counts of chicken feed inoculated with 3 log CFU/g decreased from 3.8 to 3.0 log CFU/g within a time frame of 6 days. The chicken feed used had a high moisture content (circa 50%), and it was incubated at 27°C and 60% relative humidity, which seemed favorable for S. aureus growth (65). However, the decrease might be due to a dilution effect caused by the addition of noninoculated substrate on days 2 and 4 during rearing, according to the feeding protocol used. The inoculation level of 7 log CFU/g appeared to be sufficient for S. aureus to overcome the dilution effect and to remain present during the 6 days. The larvae were able to eradicate the pathogen completely in their interior within 6 days, even when a high inoculation level of 7 log CFU/g was applied. At the same time, the S. aureus counts in the substrate at days 2 and 6 were always lower under the conditions with larvae than without. In another inoculation trial (66), BSFL were shown to inhibit S. aureus in pig manure as well. In that study, S. aureus counts were reduced from 5.47 to 2.31 log CFU/g in 8 days in the presence of BSFL. When no larvae were present, the counts increased to about 6.5 log CFU/g. Pathogen reduction of naturally present S. aureus in chicken, pig, and cow manure has been noticed as well in the presence of BSFL (32). However, larvae were not able to reduce S. aureus counts in sterilized feed (66). Moreover, autoclaved diets did not promote larval growth (67). These last two observations not only indicate that the microbiota of the substrate is important for the intestinal bacterial community homeostasis of BSFL but also that specific microorganisms might play a role in determining the level of S. aureus, for example via competition in the substrate or via a direct negative impact on S. aureus. The latter option is discussed in more detail further on.

Even though all S. aureus counts of the larvae declined under the detection limit at day 6, the possible presence of enterotoxins produced by S. aureus cannot be excluded. These toxins can be present in the rearing substrate or in the larvae. Even though the pathogen was not detected in the larvae at the first and last day of the condition with an inoculation level of 7 log CFU/g, it was detected at day 2 in the larvae. To the authors’ knowledge, there are no data available on the possible presence of enterotoxins produced by S. aureus in BSFL.

Notwithstanding the high suppression of S. aureus in our experiments, the presence of S. aureus in the rearing environment may imply a food safety risk. It is still crucial to implement good hygiene and monitoring practices in rearing facilities. In future inoculation trials, other rearing protocols and other substrates than chicken feed (for instance, organic waste streams like chicken litter or tomato leaves) that naturally harbor S. aureus can be included to support appropriate risk assessments. The expression of antimicrobial peptides in BSFL is diet dependent (68), so pathogen dynamics during rearing using other substrates may differ and should not be generalized.

### Potential mechanisms resulting in the reduction of Staphylococcus aureus counts.

It is known that BSFL thrive on substrates with a high bacterial load, such as animal and human manures (1). Moreover, it has been shown that BSFL are able to compete with S. aureus, reducing their number (32, 66). The exact mechanisms resulting in the S. aureus suppression in these studies and in the inoculation trials executed in this study are not clear. However, these studies suggest that BSFL and/or the intestinal microbiota produce potent antimicrobial agents. To this end, 178 isolates from a representative collection of the most abundantly present microorganisms from BSFL generated in previous research (24) were investigated. The isolates were screened *in vitro* for their antimicrobial activity against S. aureus. Six *Trichosporon* spp. isolates showed a substantial *in vitro* inhibitory effect, as shown in Figure 2. *Trichosporon* spp. have already been associated with industrially reared insects (69) and even with BSFL (70). Moreover, *Trichosporon* spp. are even identified as a major part of the mycobiota of BSFL (42). Interestingly, from five groups of BSFL fed on two different diets (chicken feed or vegetable waste), *Trichosporon* was the most abundant genus (87.7%) found in the larval group grown on chicken feed (42). In fact, four of the six isolates of *Trichosporon* spp. used in this study were indeed collected from BSFL fed on chicken feed substrate.

Currently, research is focusing on insects and their microbiota in the search for new antimicrobials, such as antimicrobial peptides (AMPs) (71). So far, two different fungal species isolated from the BSFL gut with antimicrobial activity have been described in the literature; Chrysosporium multifidum exhibits moderate activity against S. aureus (72), and Trichosporon asahii was found to inhibit the growth of the yeasts Candida glabrata and Candida lusitaniae (42). *Trichosporon* is known as a “killer yeast” and produces the antifungal AMP oranicin P16 (42, 73, 74). However, activity against S. aureus was, up until this study, not yet demonstrated. Because the collection of isolates tested in this study is representative for BSFL, it can be postulated that *Trichosporon* species were present during the inoculation trials and contributed to S. aureus reduction.

Next to the larval microbiota, the larvae themselves can play a role in the suppression of S. aureus. It is known that BSFL have a robust immune system (75) and can express a broad spectrum of AMPs as well (68, 75–81). In fact, a wide range of these peptides obtained from BSFL are currently characterized (68, 75–81), and six of them exhibit inhibitory activity against S. aureus (76, 77, 79–83). Although in the literature, *in vitro* inhibitory activity against S. aureus has been observed for these antimicrobial peptides, it is not known whether these peptides were induced during the inoculation trials and, if they were present, what role these peptides play in the S. aureus suppression noticed in this study. Nevertheless, the expression of the AMPs is triggered when the larvae are fed with substrates containing a high bacterial load (68), suggesting that this also might have been the case in our study. Further, the immune genes dual oxidase (*BsfDuox*) and Toll-like receptor 3 (TLR3) (*BsfTLR3*) are considered key factors in the suppression of pathogenic bacteria (66). In addition, high lysozyme activity has been found in the larval gut, catalyzing the lysis of Gram-positive bacteria (84). However, it can be questioned whether lysozyme is involved in the reduction of S. aureus in BSFL, since pathogenic S. aureus is described to be lysozyme resistant (85, 86). In the end, it is highly likely that the sum of all the interactions between the larvae, microbiota, and substrate eventually leads to the observed pathogen reduction.

## CONCLUSION

This study gained information on the general microbiological quality and the occurrence of S. aureus in a range of organic waste streams as potential substrate ingredients for BSFL rearing. The food pathogen S. aureus appeared to be very abundant in organic waste streams, with values up to almost 9.0 log CFU/g. Water activity and pH were considered to be limiting factors for S. aureus growth. Hence, insect producers should pay attention when mixing waste streams, since suitable environmental conditions can be created for the pathogen to grow again. In the inoculation trials, S. aureus counts of the substrate were always lower when larvae were present than when no larvae were present. Further, S. aureus counts of the larvae were below the detection limit (2.0 log CFU/g) after 6 days, regardless of the inoculation level. Hence, our study revealed that BSFL exert a reducing effect on S. aureus counts of both substrate and larvae. Future studies should include other rearing protocols and substrates to explore if this effect is general or substrate/protocol dependent. This study also explored the antimicrobial activity of dominant microorganisms associated with BSFL as one of the possible mechanisms regulating S. aureus load. While 58% of the 178 isolates showed signs of activity against this pathogen, the highest activity was observed for a *Trichosporon* species. This might be a new example of an insect-associated microorganism playing a role in the control of specific microbes during the rearing process of BSFL.

## MATERIALS AND METHODS

### Waste stream sampling and sample preparation.

Ten different organic waste streams were collected at different locations. Chicken litter was obtained from a local broiler farm (Proefbedrijf Pluimveehouderij, Geel, Belgium) and consisted of chicken excreta, spilled chicken feed, feathers, and bedding material. Vegetable auction overproduction consisted of unsold tomatoes, lettuce, cauliflower, and cucumber and were obtained from a fruit and vegetable auction (BelOrta, Sint-Katelijne-Waver, Belgium). Corn meal, grain mix, apple pulp, and fruit puree, which were the residual streams of several industrial production processes, were obtained from an animal feed producer (Duynie, Nijmegen, the Netherlands). Catering/supermarket/industrial food waste and household food waste were obtained from a waste management company (Renewi, Kampenhout, Belgium), where the food waste had been removed from its package and mixed into a slurry. While the catering/supermarket/industrial food waste was collected by the waste management company itself, the household food waste was collected by a local specialized waste-collecting company (Limburg.net, Hasselt, Belgium). Finally, strawberry leaves and tomato leaves were obtained from a local composting company (Metrans Vermeiren, Meer, Belgium), where leftover leaves were collected, shredded, and sieved after strawberry or tomato harvesting at horticulture companies.

Each waste stream, except strawberry and tomato leaves, was sampled twice at two different times. Sampling period 1 ranged from June to September, whereas sampling period 2 ranged from January to February in the year after sampling period 1. Because of their seasonal dependency, strawberry and tomato leaves were only obtained once in July or September, respectively. All waste streams were stored maximally 24 h at 4°C before analysis. Three replicates were taken randomly at different places in the waste stream recipients. For vegetable auction overproduction, a mixture of tomato, lettuce (including sand and root), cauliflower (with leaves), and cucumber was prepared in a 2.5:2:1:2 ratio with a home-type kitchen mixer (Ergomixx, Bosch, Gerlingen, Germany).

### Analysis of intrinsic parameters of waste streams.

All organic waste streams of periods 1 and 2 were subjected to measurement of pH using a digital pH meter (Portamess 911, Knick, Berlin, Germany, with an SI Analytics electrode, Mainz, Germany) at room temperature. Based on the method of a previous study (38), for dry samples, 17 ml of demineralized water was added to 10 g of the sample before pH measurement. For waste streams sampled in sampling period 2, water activity (*a*_w_) and moisture content were also determined. Water activity measurements were performed using a water activity meter (LabMaster *a*_w_, Novasina, Lachen, Switzerland) as soon as the water activity and temperature (25°C) were stable for 5 min. Moisture content was determined by calculating the weight loss of 5 g of the sample after overnight oven drying at 105°C. All measurements of pH, *a*_w_, and moisture content were performed in triplicate.

### Experimental setup for inoculation trials with Staphylococcus aureus.

**FIG 4:**
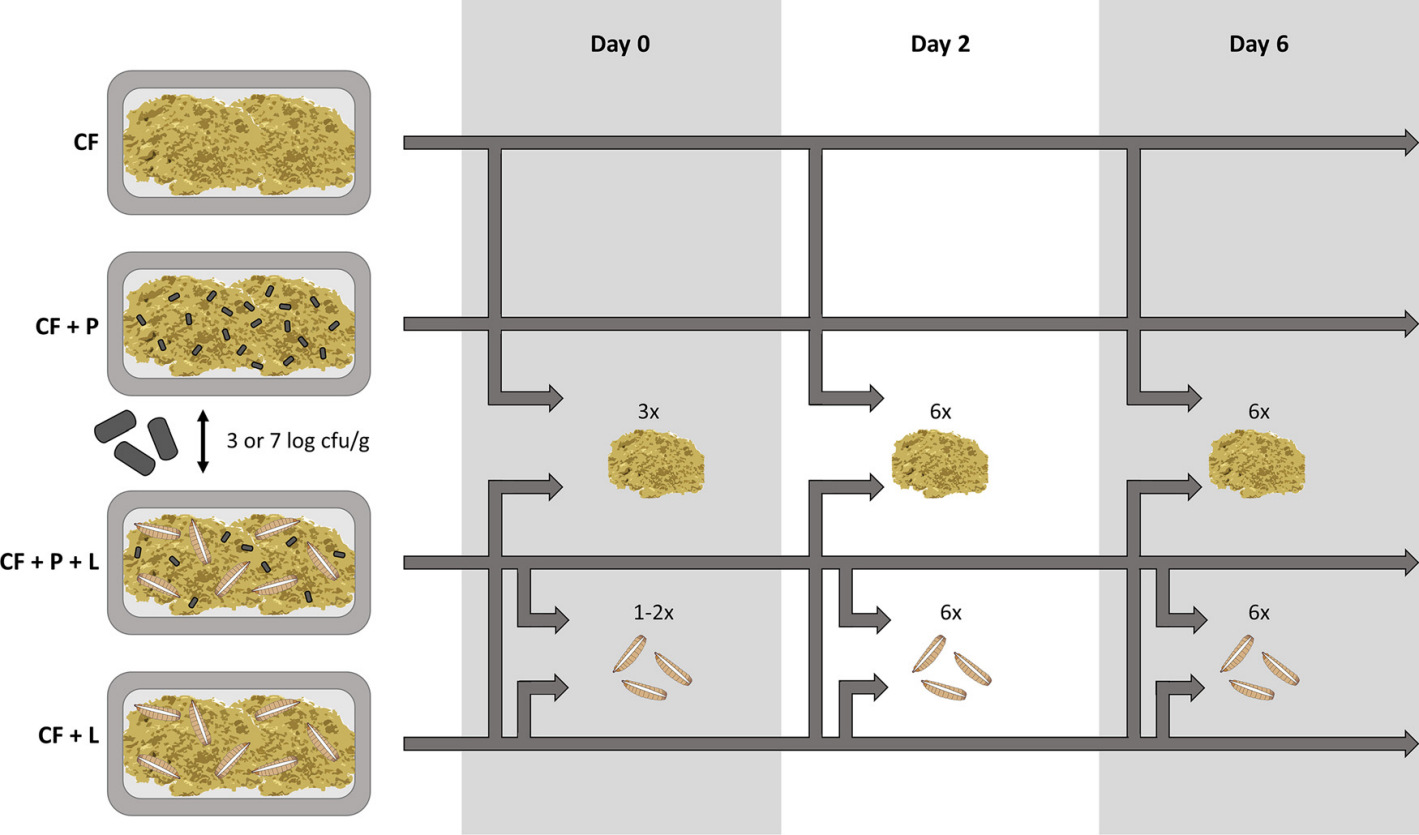
Experimental setup of the inoculation trial (CF, chicken feed; P, pathogen Staphylococcus aureus; L, larvae).

To perform inoculation trials, chicken feed was inoculated with a Staphylococcus aureus strain to assess the fate of the food pathogen and its potential transmission to BSFL reared on this substrate. The experimental setup, visualized in Fig. 4, consisted of six conditions at laboratory scale, including noninoculated chicken feed without larvae (referred to as CF), noninoculated chicken feed in the presence of larvae (CF + L), chicken feed inoculated with S. aureus and without larvae (CF + P), and chicken feed inoculated with S. aureus and with larvae (CF + P + L). For the conditions where S. aureus was inoculated in the chicken feed, two different inoculation levels were included, aiming to reach levels of 3 and 7 log CFU/g substrate at day 0. On that day, 8-day-old larvae were added to the chicken feed under the conditions where larvae were needed. For each condition, the larvae originated from a different batch of BSFL, meaning that the larvae were produced at a different time and hence in another rearing cycle. For each condition, six replicates were performed, and conditions were incubated separately, since airborne contamination between the different conditions was noticed in preliminary tests.

### Staphylococcus aureus cultivation and substrate inoculation.

Due to the high abundance of a background microbiota on the selective plates as observed in preliminary experiments not described here, a kanamycin-resistant S. aureus strain SA113 (85) was used in all experiments described in this paper. The strain was grown overnight in Luria-Bertani broth (LB; 10.0 g/L peptone [Biokar Diagnostics, Beauvais, France], 5.0 g/L yeast extract [VWR, Leuven, Belgium], and 5.0 g/L NaCl) in the presence of kanamycin (30 μg/mL; Thermo Fisher Scientific, Merelbeke, Belgium) at 30°C on a shaking plate (Orbital mini shaker, VWR) at 150 rpm. Next, the turbid culture was centrifuged at 3,060 × *g* for 5 min (Multifuge 3S, Heraeus, Hanau, Germany), and the resulting supernatant was removed. Then, the pellet containing the bacterial cells was resuspended in peptone physiological salt solution (0.85% NaCl, 0.1% peptone; Biokar Diagnostics). The prepared inoculum was further diluted with peptone physiological salt solution until either 2.0 or 4.6 McFarland units (MFU) was reached using a densitometer (DEN-1 McFarland densitometer, Grant Instruments, Cambridge, UK). To reach 3 or 7 log CFU/g chicken feed, 1.70 mL of a 1/2,000 diluted MFU 2.0 solution or 1.64 mL of an MFU 4.6 solution, respectively, were needed. In preliminary tests, the S. aureus solutions of different MFUs were plated on VJA (Sigma-Aldrich, Overijse, Belgium) supplemented with a 1% potassium tellurite solution (20 mL/L VJA, Sigma-Aldrich) (87) to define the solutions needed.

For the substrate, chicken feed (Startmeel voor Kuikens 259, AVEVE, Geel, Belgium) was selected to standardize the experimental conditions as much as possible. It was ground with a mixer (EP9800 Powerblender, Espressions, Eindhoven, the Netherlands) using the “Ice Crush” program twice. The ground chicken feed was mixed with tap water in a 1:1 ratio (wt/vol), and, per replicate, 100 g of this mixture was placed in polypropylene trays (1 L). Next, the proper aforementioned aliquot of the prepared inoculum was added to the corresponding condition. To the noninoculated substrate, 1.70 mL of sterile peptone physiological salt solution was added. Both solutions were homogenized in the chicken feed using a sterile spoon.

### BSFL rearing and sampling.

The 8-day-old BSFL used in this study were obtained from a laboratory colony reared by RADIUS (Thomas More University College, Geel, Belgium). The larvae-rearing methods until day 8 are described in a previous study (24). At the start of each inoculation trial, 100 g of (non)inoculated substrate per replicate was placed in a container (10 cm × 15 cm) and circa 500 8-day-old BSFL (as determined by the average weight of 5 × 10 larvae) for the conditions with larvae, leading to a larval density of 3.3 larvae/cm^2^. All containers were covered with a lid with mesh and placed in a climate chamber (Pharma 600, Weiss Technik, Liedekerke, Belgium) at 27°C and 60% relative humidity. On days 2 and 4, an additional 100 g of chicken feed mixed with tap water was added to the containers. This feed was uninoculated for all conditions.

Samples were aseptically taken on day 0, day 2, and day 6 (Fig. 4). Three substrate samples were taken on day 0 after inoculation with S. aureus or physiological salt solution. At the same time, one larval sample was taken before distributing the BSFL over the separate boxes. On days 2 and 6, one sample of larvae and one sample substrate was taken per replicate. The substrate was separated from the larvae by sieving, and, to eradicate the microorganisms present on the exoskeleton, a disinfection method was applied, as described in earlier studies (23, 24). Briefly, the larvae were washed under running tap water and then subjected to three washing steps (one with 70% ethanol and two with sterile distilled water) of 1 min at 200 rpm on a laboratory shaking table (Unimax 1010, Heidolph, Schwabach, Germany). The larval mass was then homogenized with a home-type kitchen mixer (Bosch). In addition, larval growth was monitored on days 0, 2, and 6 by measuring the weight of 5 × 10 larvae picked randomly.

### Microbiological analyses.

Microbial plate counts were determined according to the ISO standards as described by reference 88. For the waste streams, a primary dilution was obtained by diluting 5 g of each sample in 45 g of sterile peptone physiological salt solution, followed by a homogenization step in a stomacher (BagMixer, Interscience, Saint Nom, France) for 1 min. For strawberry and tomato leaves, the samples were mixed together with the solution for 1 min using a home-type kitchen mixer (Bosch) before homogenization in the stomacher. A tenfold dilution series from the primary dilution was plated on different media. Total (aerobic) viable counts were determined on plate count agar (PCA; Biokar Diagnostics) and incubated at 30°C for 72 h, Enterobacteriaceae on violet red bile glucose agar (VRBG; Biokar Diagnostics) after incubation at 37°C for 24 h, and LAB on de Man Rogosa and Sharpe agar (MRS; Biokar Diagnostics) followed by incubation at 30°C for 72 h. Yeasts and molds were determined after streak plating on dichloran Rose Bengal chloramphenicol agar (DRBC; Biokar Diagnostics) and incubation at 25°C for 5 days. Aerobic bacterial endospores were determined by subjecting the 10^−1^ dilution to a heat shock (80°C for 10 min), followed by preparation of a ten fold dilution series, plating on PCA, and incubation at 37°C for 24 h. Iron sulfite agar (ISA; Oxoid, Thermo Fisher Scientific) was used to determine sulfite-reducing clostridia after anaerobic incubation at 37°C for 48 h. Anaerobic conditions were generated in 2.5 L anaerobic jars (VWR) using anaerobic gas-generating sachets (Oxoid Anaerogen 2.5 L Sachet, Thermo Fisher Scientific) and evaluated using resazurin anaerobic indicator strips (Oxoid, Thermo Fisher Scientific). Furthermore, the food pathogen Salmonella was counted after streak plating on Rapid’*Salmonella* agar (Bio-Rad Laboratories, Temse, Belgium) and incubating at 37°C for 24 h. Determination of S. aureus was performed by streak plating on VJA supplemented with a 1% potassium tellurite solution (20 mL/L VJA) and incubating at 37°C for 24 h. All microbial counts were expressed in log CFU/g. For each waste stream, three replicates were analyzed per sampling period, and plating was performed in duplicate to calculate the mean and standard deviation.

For each sample from the inoculation trial, total (aerobic) viable counts and S. aureus counts were determined in the same way as for the waste stream samples, but next to a 1% potassium tellurite solution, VJA was also supplemented with kanamycin (30 μg/mL VJA). During preliminary tests not described in this paper, it was noticed that, despite the addition of kanamycin, a background microbiota (presumably molds) was still present on the selective VJA plates. To reduce the background microbiota and to properly count the S. aureus colonies, dichloran (Sigma-Aldrich) was added in a concentration of 25 μg/mL (89) as an inhibitor for mold spreading. In contrast to the waste stream samples, VJA plates were incubated for 2 days because of the slower growth of S. aureus due to the addition of kanamycin.

### Antimicrobial activity screening of dominant microorganisms of BSFL.

In previous research (24), a representative culture collection of 172 dominant aerobic bacteria from BSFL was established via an approach based on only considering the highest serial dilutions of BSFL extracts. During the isolation process, six fungal isolates were collected as well. As described previously (24), these isolates were all subjected to random amplification of polymorphic DNA (RAPD), leading to two distinct patterns, with five of the fungal isolates characterized by the same pattern and one fungal isolate characterized by a slightly different pattern. This last isolate and three fungal isolates from the other RAPD pattern were then identified via amplification and sequencing of the fungal internal transcribed spacer (ITS) region. The ITS region was amplified using the primers ITS5 (5′-GGA AGT AAA AGT CGT AAC AAG G-3′) and LR3 (5′-CCG TGT TTC AAG ACG GG-3′) (90) and sequenced at Eurofins Genomics (Ebersberg, Germany). The obtained sequence data were aligned by BioEdit software (91), and a BLAST search in GenBank was performed to identify the four isolates. The remaining two isolates were identified according to the corresponding RAPD pattern, as explained in reference 24. All 178 microorganisms of the collection were subjected to antimicrobial activity screening against S. aureus (BCCM LMG 8064) using a modified deferred growth inhibition assay (25) in the version of reference 92. Briefly, a colony of each isolate, grown overnight on a PCA plate at 30°C, was spotted on a new PCA plate at a ratio of four isolates per plate and subsequently incubated for 48 h at 30°C. After incubation, an overnight culture of S. aureus grown in LB broth and shaken at 150 rpm at 37°C was adjusted to an MFU of 4.0 and added to the agar plates using a soft agar overlay technique; soft LB agar 0.75% (wt/vol) was inoculated with S. aureus at a concentration of 5% (vol/vol) and added on top of the isolate spots. After solidification, the agar plates were incubated for another 24 h at 37°C. Afterward, the presence of any inhibition zones was observed. For the isolates with clear activity, that is, a large size of inhibition zone and/or high clarity, the radii of the inhibition zones were measured starting from the center of the colony. Antimicrobial activity screening was performed twice.

### Statistical analyses.

To determine statistical differences in intrinsic factors between waste streams, one-way analysis of variance (ANOVA) was used, followed by a Tukey’s honestly significant difference (HSD) *post hoc* test. Normality and homoscedasticity assumptions were evaluated using a Shapiro-Wilk test and Levene’s test, respectively. If a normal distribution was not confirmed, a nonparametric Kruskal-Wallis analysis was performed, followed by a Wilcoxon each pair test. To determine statistical differences in microbial counts and pH between sampling periods 1 and 2 for each waste stream, results were subjected to an independent samples *t* test. If a normal distribution was not confirmed, a nonparametric Mann-Whitney U test was performed, and Welch’s *t* test was used in case of unequal variances. Statistical differences in means for the total viable counts and the S. aureus counts for the inoculation trial were compared using one-way ANOVA followed by a Tukey’s HSD *post hoc* test. With unequal variances, Welch’s ANOVA with a Steel-Dwass all pairs *post hoc* test was used. If counts were below the detection limit, the detection limit itself was used for statistical analysis. All tests were performed with the JMP Pro 15.0.0 software package (SAS Institute Inc., Cary, NC, USA). A significance level of *α* = 0.05 was considered for all statistical analyses.
